# An Analytical Symptomatology of the Homœopathic Materia Medica

**Published:** 1892-08

**Authors:** 


AN ANALYTICAL SYMPTOMATOLOGY OF THE HOMŒOPATHIC MATERIA MEDICA, by Rufus L. Thurston, M. D., and Samuel A. Kimball, M. D. Boston, Mass. Printed by Wm. B. Libby, 16 Arlington Street. 1892.

This publication consists of a fascicle of twenty-four pages of a new materia medica that is designed for use at the bedside. Our readers will remember seeing an announcement of it in the May number, at page 223. The present issue contains Abrotanum, Absinthum, Acetic Acid, and part of Aconite. Every homœopathic physician will need it and its issue should be hastened by all subscribing for it. Subscriptions may be sent to Dr. S. A. Kimball, 124 Commonwealth Avenue, Boston, Massachusetts.

